# Supplementary material for: Alternative splicing links histone modifications to stem cell fate decision
Source: Genome Biol. 2018 Sep 14;19:133. doi: 10.1186/s13059-018-1512-3 (PMC6138936; doi:10.1186/s13059-018-1512-3)
Supplement: Supplementary file 1 — Figure S1. Identifying hESC differentiation-related AS exons. Figure S2. The hESC differentiation-related AS exons possess the typical properties of AS exons. Figure S3. AS profiles upon hESC differentiation show lineage-specific splicing pattern. Figure S4. HMs change significantly around the alternatively spliced exons upon hESC differentiation. Figure S5. A subset of AS events are significantly associated with some HMs upon hESC differentiation. Figure S6. K-means clustering based on selected epigenetic features of eight HMs for MXE and SE AS exons. Figure S7. HM-associated AS genes are more lineage-specific. Figure S8. HM-unassociated AS genes are enriched in G1 cell-cycle phase and pathways for self-renewal. Figure S9. Isoform switch from PBX1a and PBX1b during hESC differentiation. Figure S10. Isoform switch of PBX1 links H3K36me3 to hESC fate decision. Figure S11. The effect of ΔPSI cutoffs for AS-HM correlations. Table S1. The number of all AS events identified during hESC differentiation. Table S5. The PCR primers used in this study. (PDF 1917 kb) [file 13059_2018_1512_MOESM1_ESM.pdf]

Supplementary Figures S1-S11 and Tables S1, S5

Alternative splicing links histone modifications to stem cell fate decision

Yungang Xu[1,2], Weiling Zhao[1,2], Scott D. Olson[3], Karthik S. Prabhakara[3], Xiaobo Zhou[1,2*]

[1]Center for Computational Systems Medicine, School of Biomedical Informatics, The University of Texas Health Science Center at Houston, TX, 77030, USA

[2]Center for Bioinformatics and Systems Biology, Wake Forest School of Medicine, Winston-Salem, NC 27157, USA

[3]Department of Pediatric Surgery, McGovern Medical School, The University of Texas Health Science Center at Houston, Houston, TX 77030, USA

*Correspondence: xiaobo.zhou@uth.tmc.edu

Keywords: alternative splicing, histone modification, embryonic stem cell, cell fate decision, cell cycle machinery

Email addresses:
YX: yungangx.xu@uth.tmc.edu
WZ: weiling.zhao@uth.tmc.edu
KP: Karthik.Prabhakara@uth.tmc.edu
SO: scott.d.olson@uth.tmc.edu
XZ: xiaobo.zhou@uth.tmc.edu

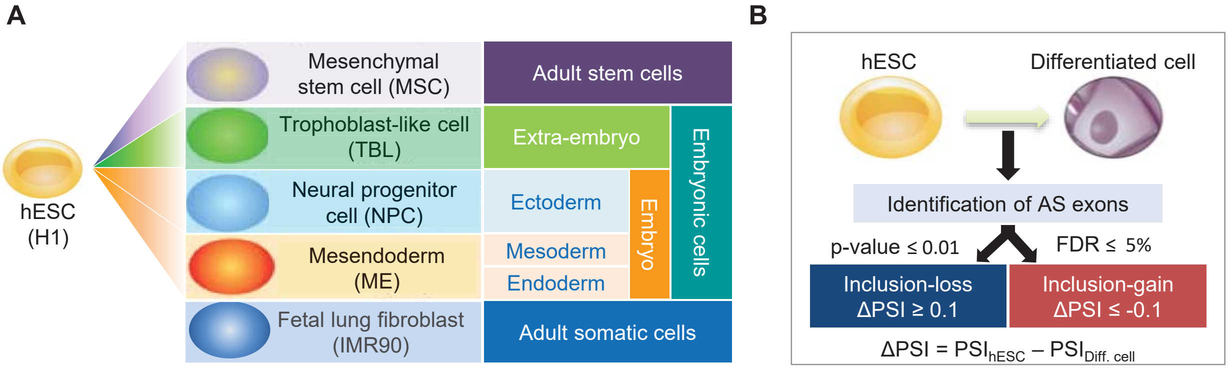

C

Mutually exclusive exon (MXE) | Skipped exon (SE)

Cell lineages	#Reads	MXE*	TF	coTF	CRF	HK	Bivalent	Gain	Loss	MXE.sp	%sp	SE*	TF	coTF	CRF	HK	Bivalent	Gain	Loss	SE.sp	%sp
→ME	770M	177	13	3	7	66	4	102	75	80	0.452	484	34	7	8	167	29	160	324	244	0.504
→TBL	709M	426	36	10	11	164	36	246	180	217	0.509	687	42	17	6	218	59	264	423	218	0.317
→MSC	775M	398	28	7	7	148	39	209	189	157	0.394	1152	77	23	10	388	97	458	694	277	0.240
→NPC	806M	589	35	13	8	210	27	344	245	360	0.611	1636	108	38	23	562	98	423	1213	969	0.592
→IMR90	319M	2583	124	50	31	1133	137	1588	995	2211	0.856	1655	98	26	18	606	118	758	897	735	0.444
H1	780M																				

*AS event numbers, of which MXE involves pairwise exons, but only the upstream exons are considered in following analysis; #Reads: the sum of reads number for 2 replicates; AS events are subject to |ΔPSI| ≥ 0.1, FDR ≤ 5%.

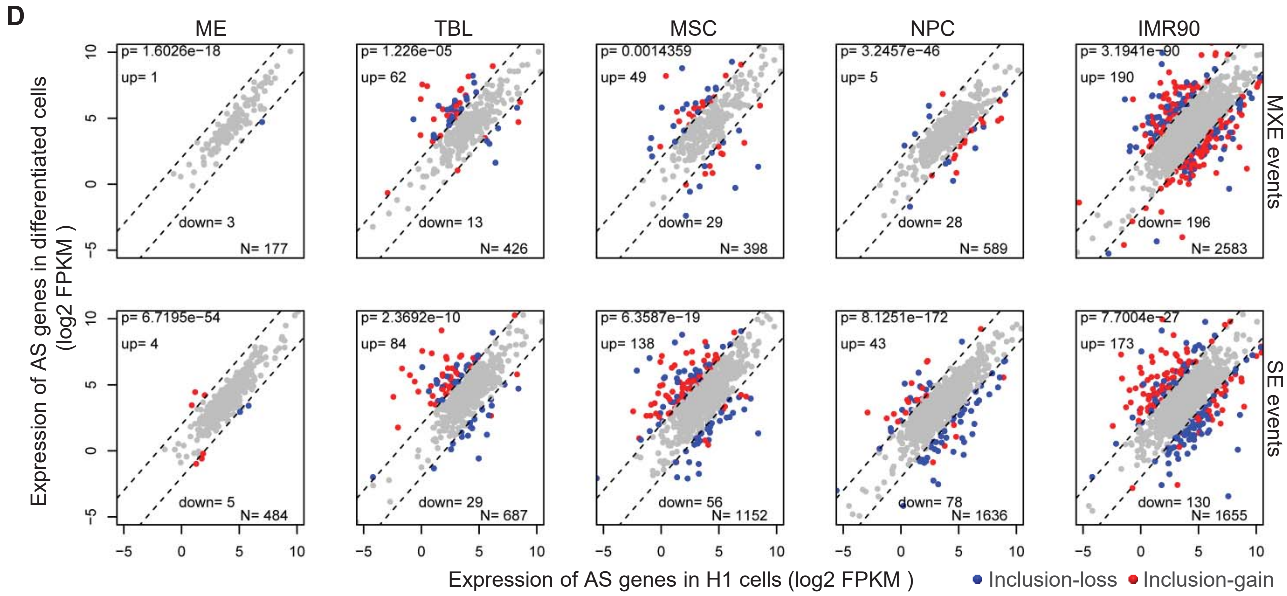

Figure S1. Identifying hESC differentiation-related AS exons. (A) The schematic of hESC differentiation and a summary of the histological relations between the six cell types considered in this study. Pair-wised comparing differentiated cells with hESCs represents multiple differentiation lineages. **(B)** The identification of AS exons upon hESC differentiation. The AS exons were identified if their changes of 'percent spliced in' (ΔPSIs) are greater than 0.1 (inclusion-loss) or smaller than -0.1 (inclusion-gain) between H1 and differentiated cells with the FDRs are less than 5%. **(C)** The statistics of hESC differentiation-related AS events. TF, transcription factor; coTF, transcription co-factor; CRF, chromatin remodeling factor; HK, housekeeping gene. Gain or loss, inclusion-gain or -loss AS events; MXE.sp and SE.sp, lineage-specific MXE and SE events, respectively; %sp, percentage of lineage-specific AS events. **(D)** The majority of genes hosting the AS exons are not differentially expressed between H1 cells and differentiated cells (fold change < 2); p-values show the significances based on hypergeometric test; N, the number of involved AS events; up or down, the number of AS events that their hosting genes are up- or down-regulated upon hESC differentiation.

Related to **Figure 1.**

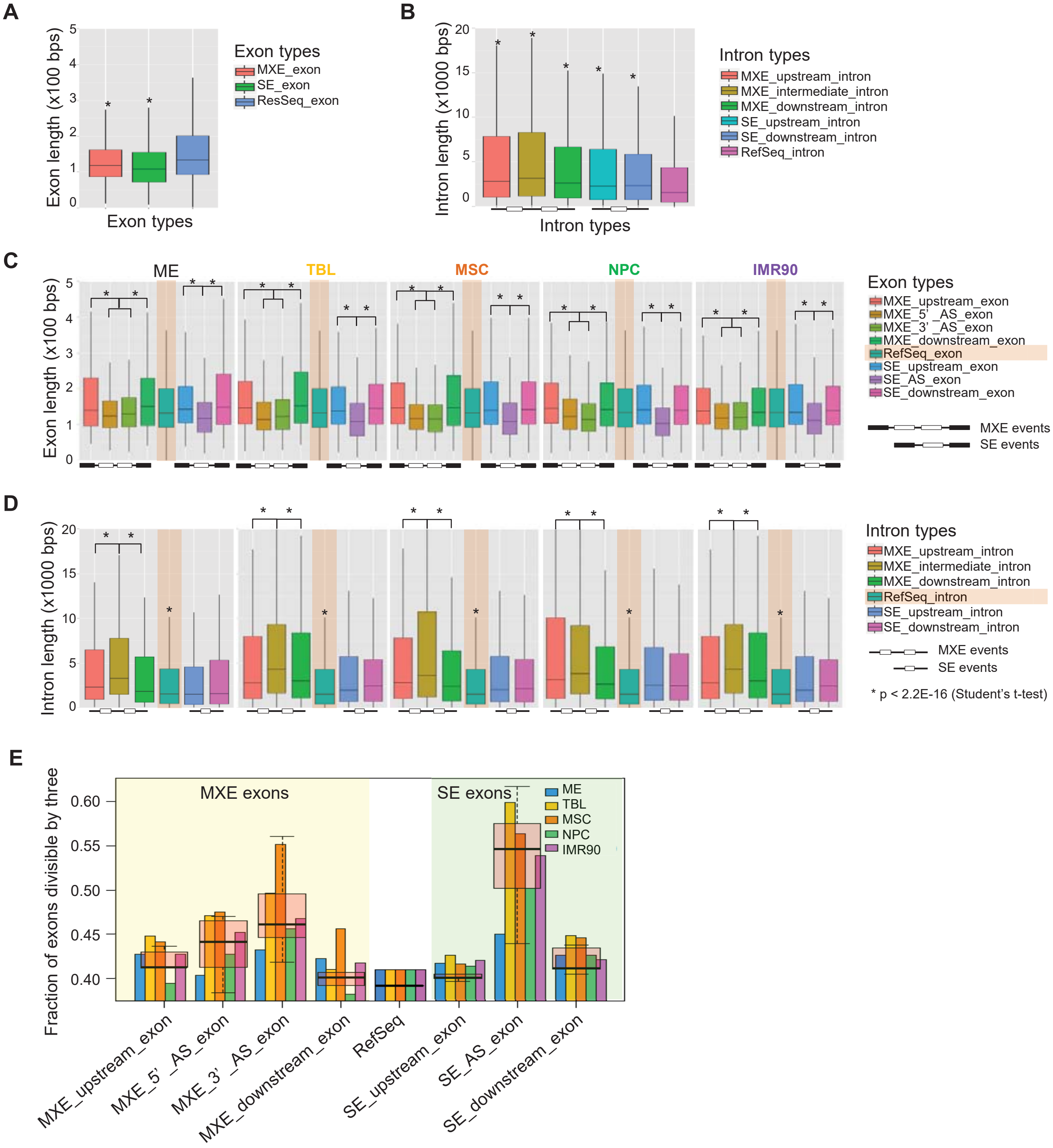

Figure S2. The hESC differentiation-related AS exons possess the typical properties of AS exons. (A) The average length of AS exons is much shorter than that of all exons based on RefSeq annotation. **(B)** The average length of adjoining introns of AS exons is much longer than that of all introns bases on RefSeq annotation. **(C)** The length of AS exons is much shorter than that of the neighboring up- or down-stream constitutive exons across all cell lineages and AS types. **(D)** The length of introns between MXE exons is much longer than that of the introns aside the AS exons and that of all introns based on RefSeq annotation. The introns surrounding SE exons are much longer than the average intron length base on RefSeq. **(E)** The length of AS exons is more often divisible by three than flanking constitutive splicing (CS) exons or all RefSeq exons. Boxplots for pooled exons of all cell types in each category.

Related **to Figure 1**.

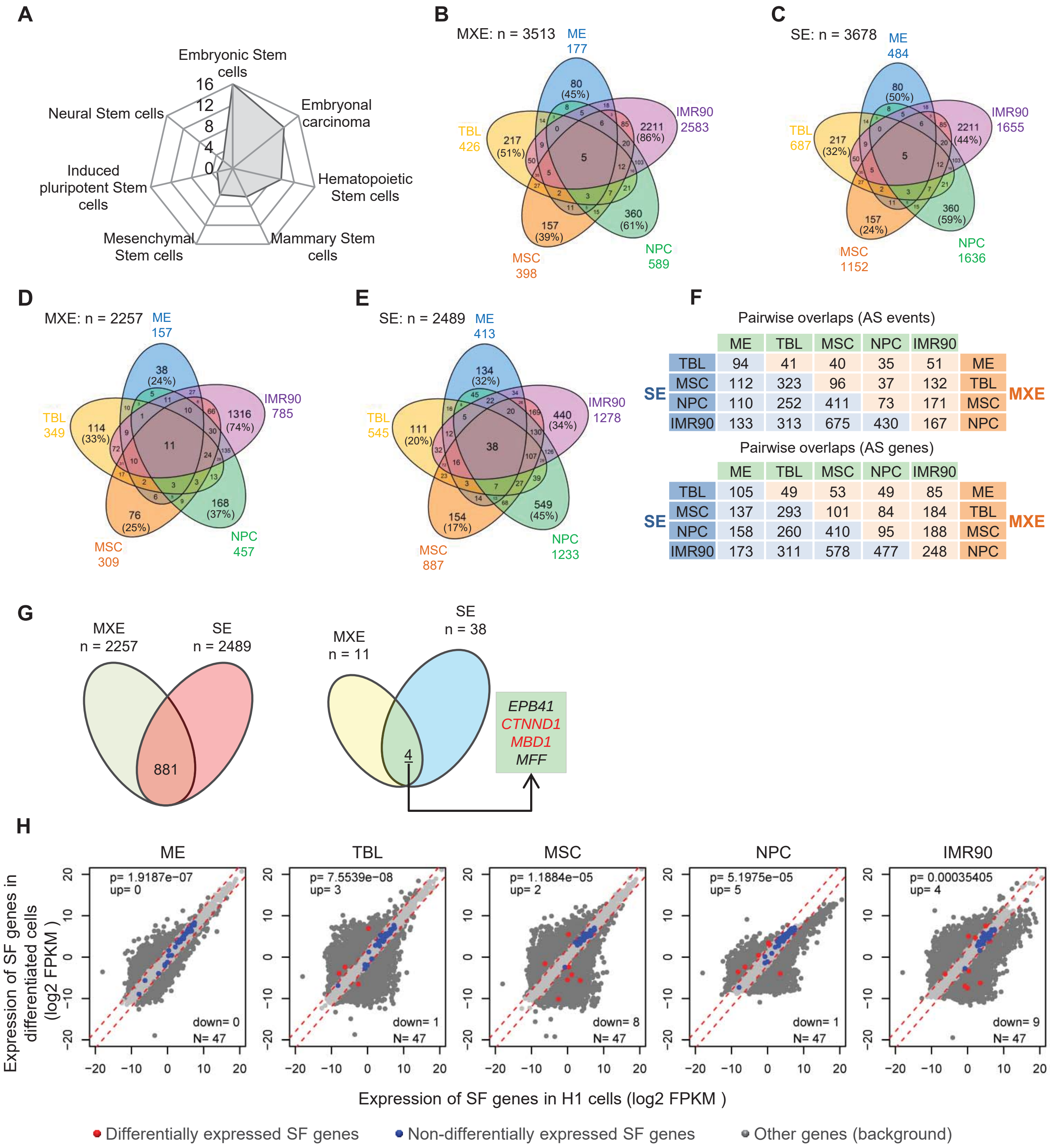

Figure S3. AS profiles upon hESC differentiation show lineage-specific splicing pattern. (A) Enrichment analysis of all AS genes based on a manually curated stemness signature gene set, showing hESC differentiation-related AS genes are significantly associated with stemness, especially, of ESCs (-log10 Bonferroni adjusted p-values). **(B)** and **(C)** Venn graphs show the overlaps of MXE and SE events between different cell lineages. Numbers indicate the count of events for each part and percentage in parentheses show the lineage-specific percentages. **(D)** and **(E)** Venn graphs showing the overlapping of MXE and SE AS genes between different cell lineages. Numbers indicate the number of genes for each part and percentage in parentheses show the lineage-specific percentages. **(F)** Pairwise overlaps of AS events (uper) or AS genes (lower) between different cell lineages. **(G)** The overlaps between SE and MXE AS genes, as well as the overlap between the inter-lineage shared MXE and SE AS genes. Genes in red have been reported that their AS events are involved in mouse ESC differentiation. **(H)** Few SF genes are differentially expressed between H1 cells and differentiated cells (fold change < 2); p-values show the significances based on hypergeometric test; N, the number of selected SF genes; up or down, the number of SF genes that are up- or down-regulated upon hESC differentiation.

Related to **Figure 1**.

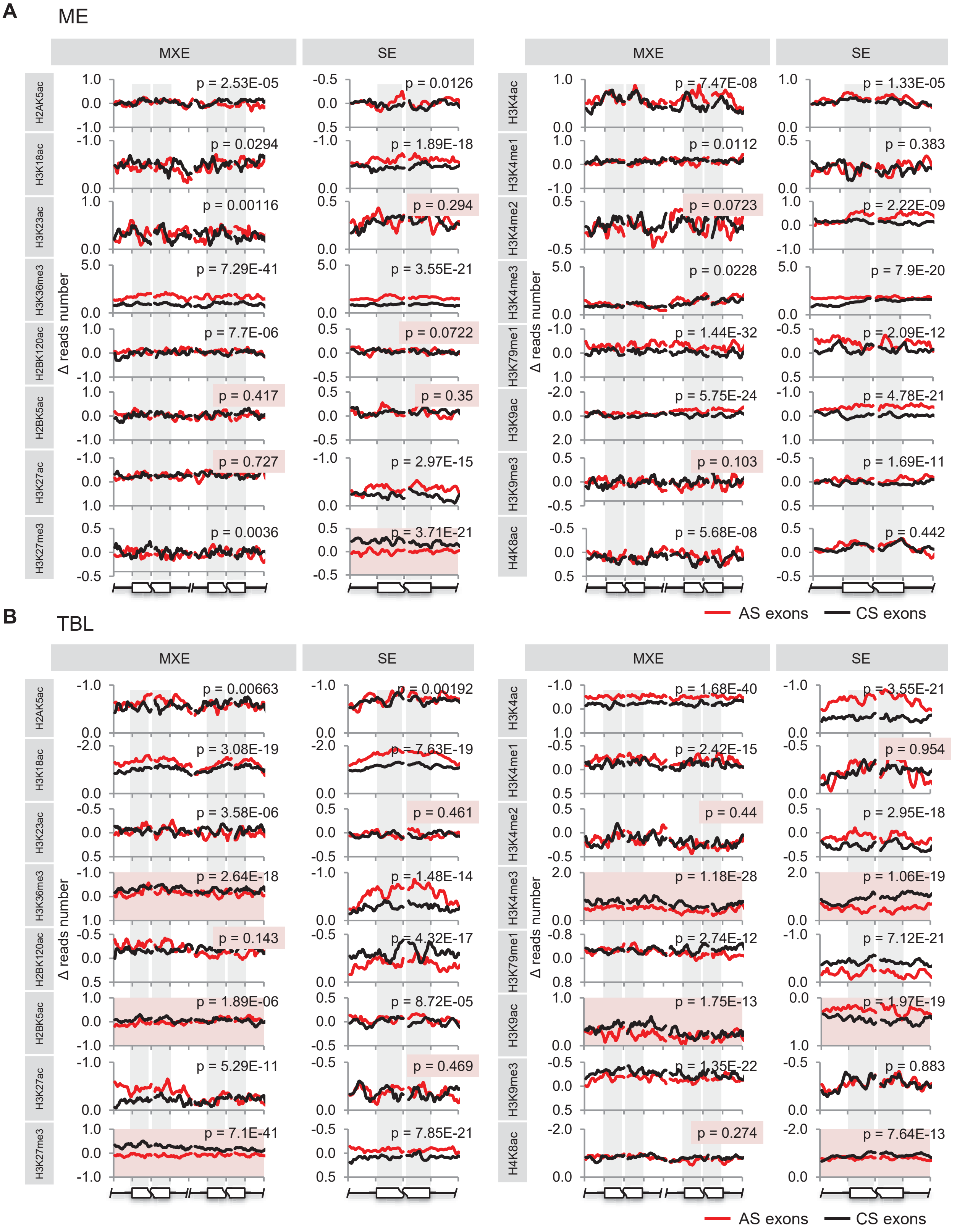

Figure S4. Histone modifications (HMs) change significantly around the alternatively spliced (AS) exons upon hESC differentiation. (A-F) The global profiles of HM changes (normalized Δ reads number) around the AS exons and randomly selected constitutive splicing (CS) exons during the differentiation from H1 ESCs to ME (**A**), TBL (**B**), MSC (**C**), NPC (**D**), or compared to IMR90 (**E**), as well as pooled them together (**F**); ±150bp regions of the splice sites (exons-intron boundaries) were considered and 15bp-binned to produce the curves. The p-values with red shading indicate no significant difference between AS and constitutive exons; Red-shaded panels indicate the cases that HMs change more significantly around constitutive exons rather than around the AS exons. p-values, Mann-Whitney-Wilcoxon test.

Related to **Figure 2**.

Figure S4 continue.

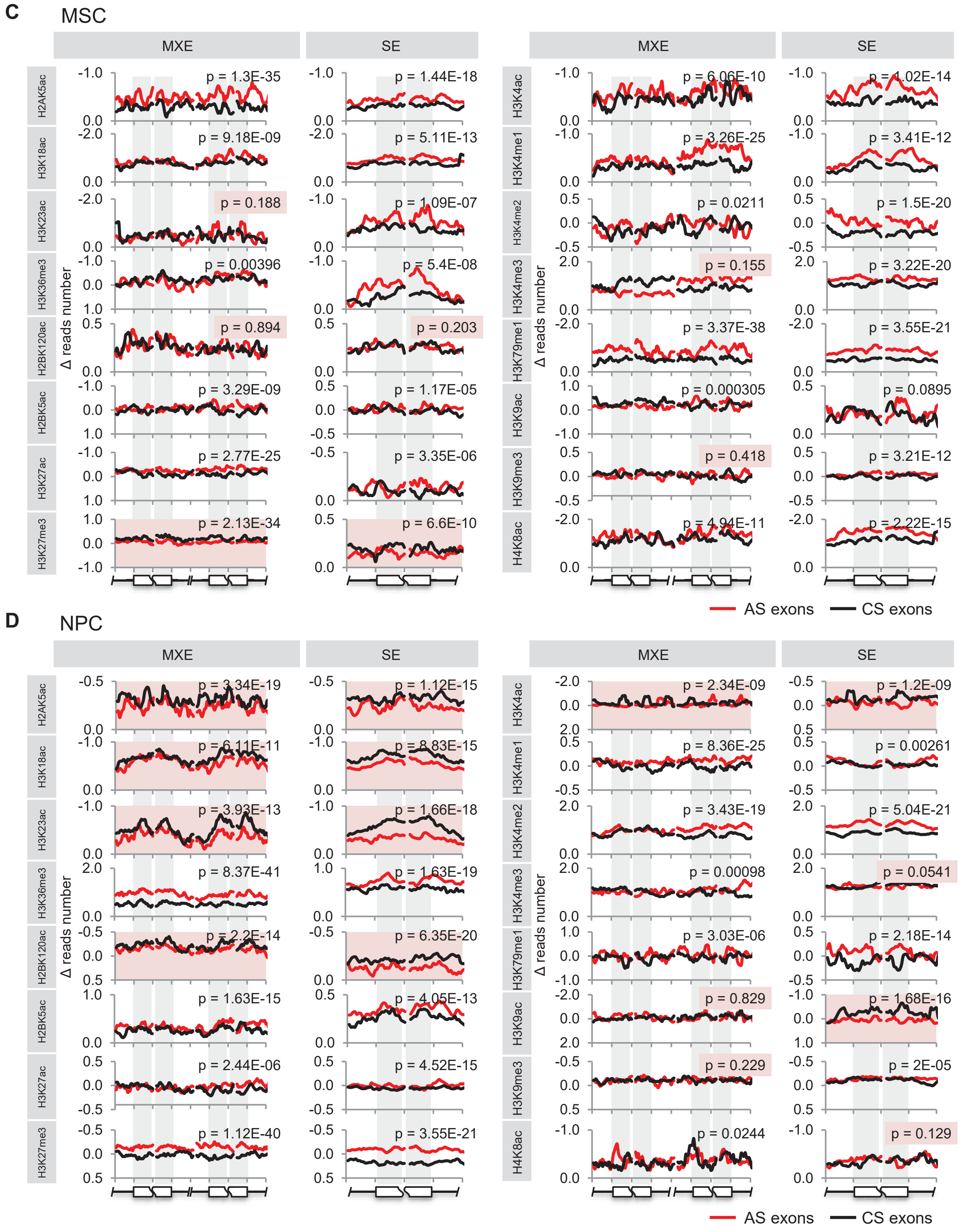

Figure S4 continue.

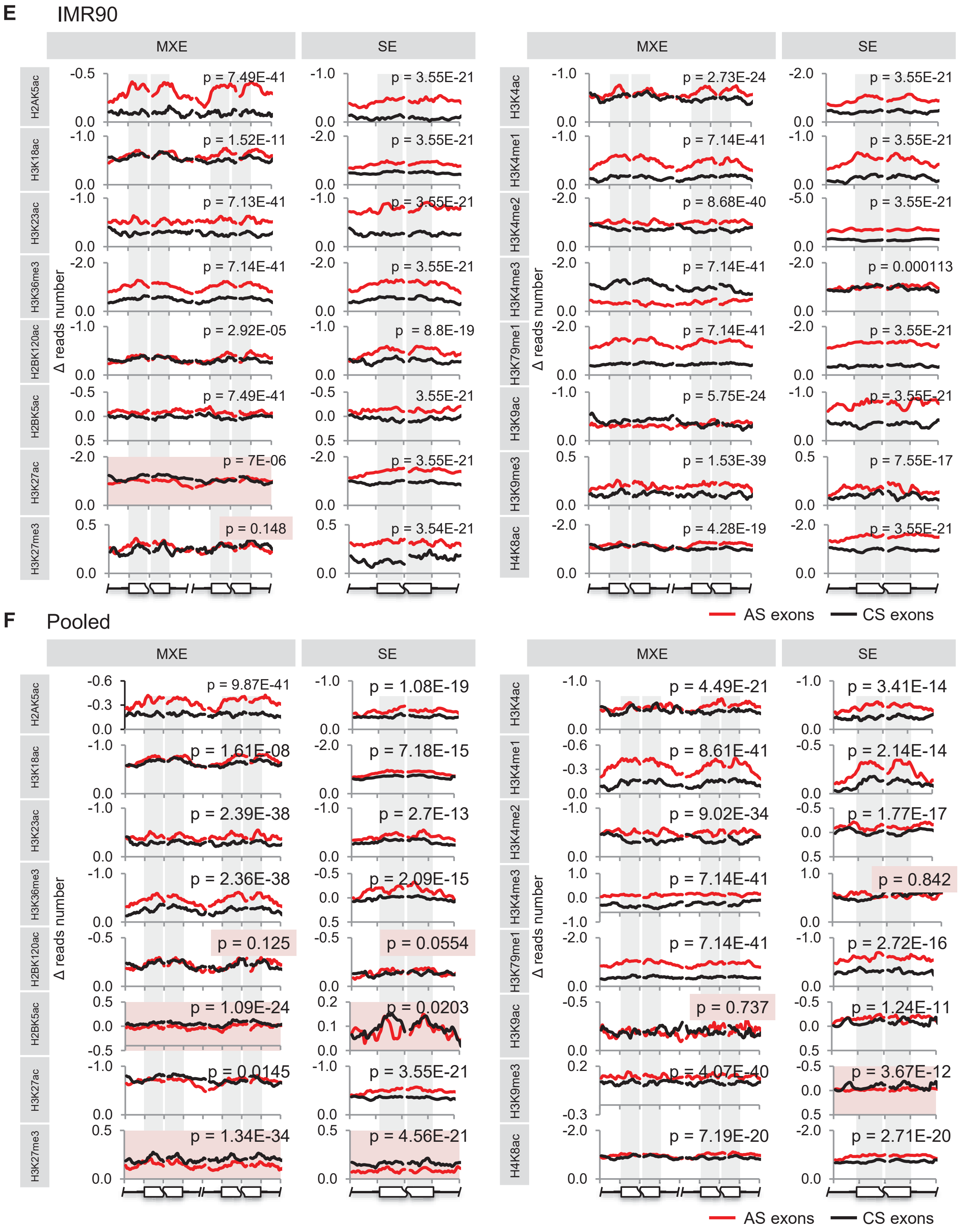

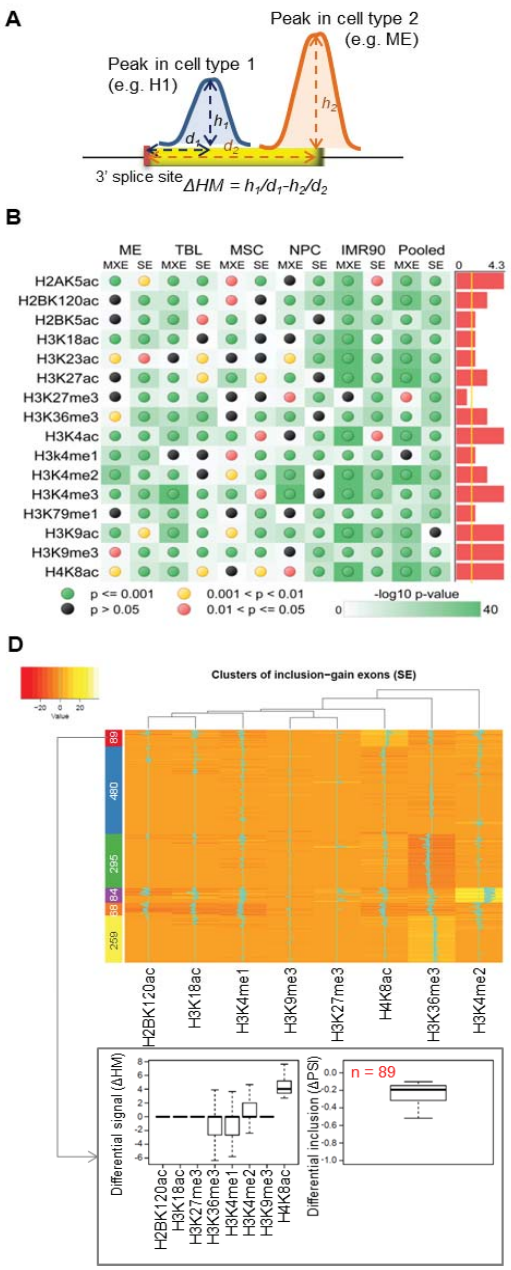

C

	MXE				SE			
	PC	MLR	LLR	Selected	PC	MLR	LLR	Selected
H2AK5ac								
H2BK120ac						0.0085	0.0206	√
H2BK5ac		0.0281		√				
H3K18ac	0.04346	0.0037		√		0.0116		√
H3K23ac								
H3K27ac		0.0086	0.0380	√				
H3K27me3					0.03921		0.0139	√
H3K36me3	0.0164	0.0025		√	2.22E-16	3.07E-09	<2E-16	√
H3K4ac								
H3K4me1	0.03104			√	0.00069			√
H3K4me2						0.0480		√
H3K4me3								
H3K79me1		0.0102		√				
H3K9ac								
H3K9me3		0.0097		√		0.0102		√
H4K8ac	0.02880	0.0172		√		0.0001		√

E

	Total AS exons	At least 1 HM changes	HM-associated AS exons
MXE	3513	3194 (90.9%)	1891 (53.8%)
SE	3678	3667 (99.7%)	1906 (51.8%)
Pooled	7191	6861 (95.4%)	3797 (52.8%)

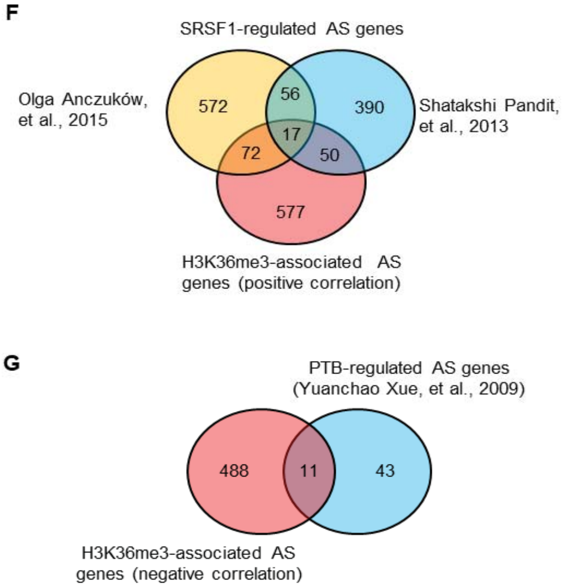

Figure S5. A subset of AS events is significantly associated with some HMs upon hESC differentiation. (A) The differential ChIP-seq signal of a HM (ΔHM) was defined as the difference of the summit heights of narrow peaks between two cell types, which were normalized by the distances (in kbs) from the peak summit to the 3' splice site. **(B)** The statistic significances of changes for all 16 HMs in each cell lineage and pooling them together (pooled), represented as the -log10 p-values based on Mann-Whitney-Wilcoxon test between the HM profiles of inclusion-gain and inclusion–loss AS exons. The side bars represent the significances whether the changes of HMs are consistently differentially enriched in inclusion-gain and inclusion–loss AS exons across cell lineages, showing the link strength between AS and HMs as the -log10 p-value based on Fisher's exact test. The yellow vertical line indicates the significance cutoff of 0.05. **(C)** Three correlation test methods were used to preselect the HMs that may associate with AS; '√' indicates the HMs passing the test ($p \le 0.05$) and will be considered for further quantitative analysis. PC, Pearson correlation; MLR, multiple linear regression; LLR, logistic regression. **(D)** A representative *k*-means clustering result of SE 'inclusion-gain' exons based on selected epigenetic features, showing one of the six clusters exhibits negative correlation between the differential H4K8ac signal and the inclusion level changes of 89 SE exons. **(E)** The statistic of the AS exons with at least one HM changes upon differentiation ($|\Delta HM| > 0$) and the HM-associated exons. The % in parentheses shows the percentage against the total AS exon number. **(F)** and **(G)** The overlaps between H3K36me3-associated AS genes and those from other literatures showing the potential mechanism by which H3K36me3 takes the role in AS regulation via proper chromatin-adapter systems. The indicated references are given in the main text.

Related to **Figure 3**.

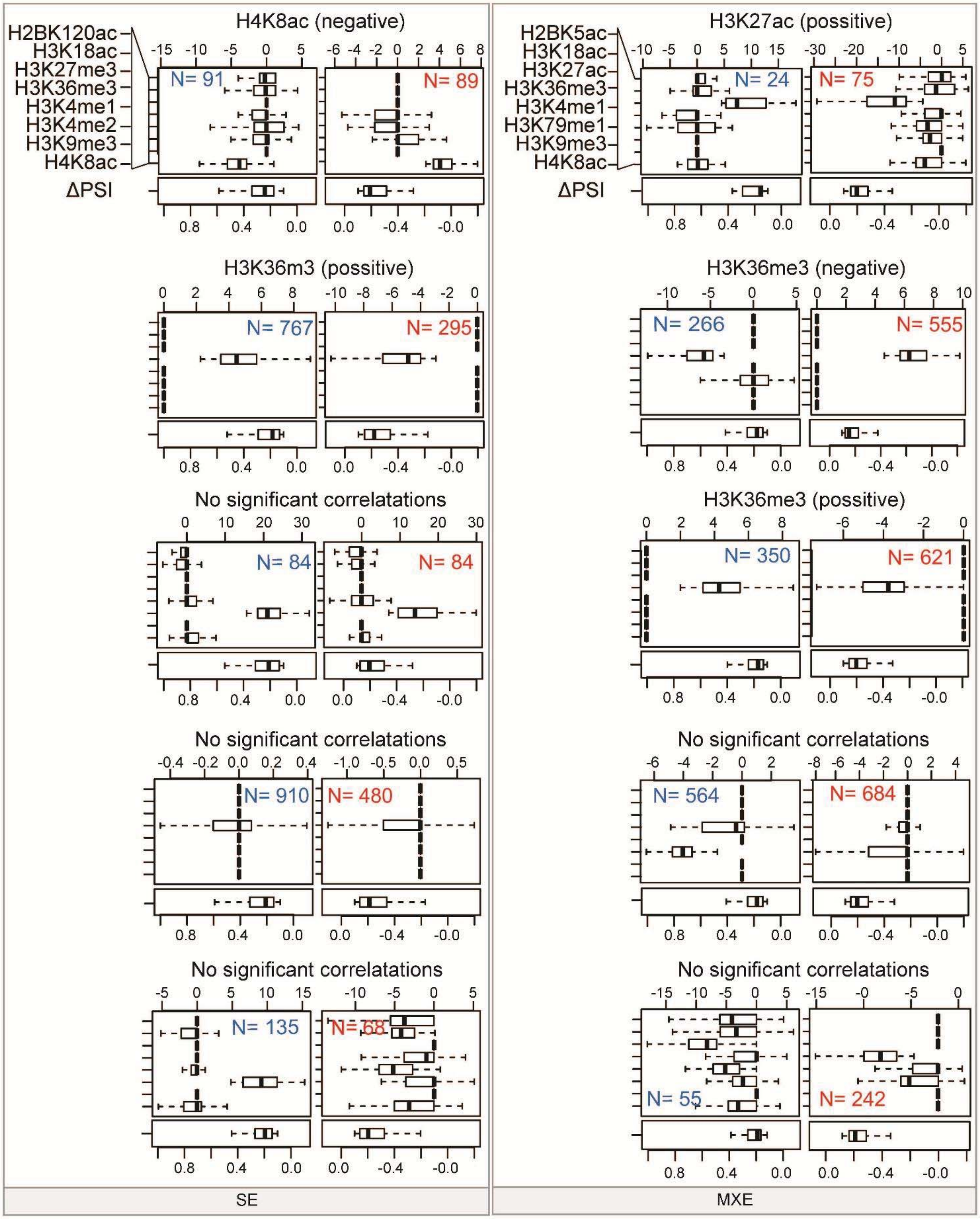

Figure S6. *K*-means clustering based on selected epigenetic features of eight HMs for MXE and SE AS exons. Each panel represents one subset of exons identified by *k*-means clustering, showing the boxplots of differential signals (ΔHMs) and corresponding differential inclusion levels (ΔPSIs). The number of exons present in each cluster is indicated in the boxplot, with blue refers to the inclusion-loss exons and red the inclusion-gain exons.

Related to **Figure 3**.

A B C
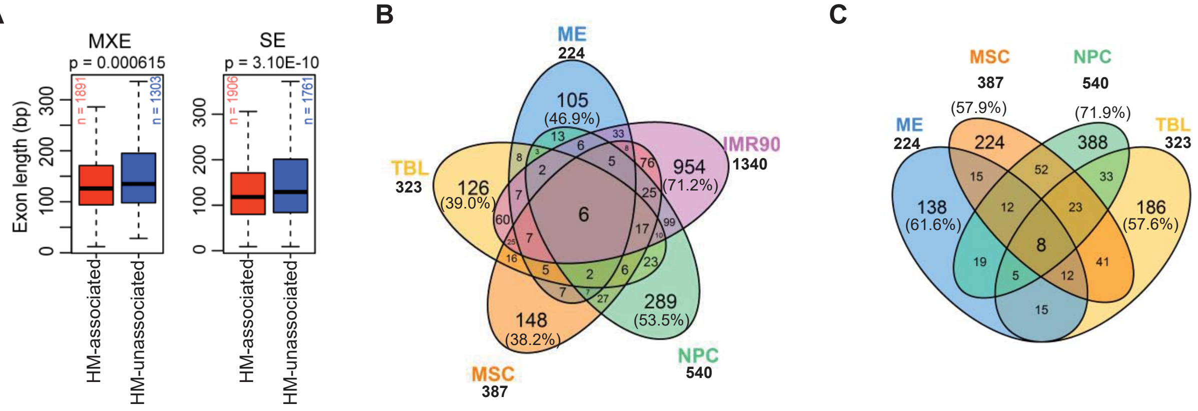

D

	ME	TBL	MSC	NPC	IMR90
ME	224				
TBL	40 (7.1%)	323			
MSC	47 (6.6%)	84 (10.0%)	387		
NPC	44 (8.7%)	69 (10.8%)	95 (12.4%)	540	
IMR90	74 (5.0%)	134 (8.4%)	169 (9.9%)	170 (11.4%)	1340

E

Name	Description	UniProtKB/Swiss-Prot function
BARD1	BRCA1 Associated RING Domain 1	This gene encodes a protein which interacts with the N-terminal region of BRCA1. Plays a central role in the control of the cell cycle in response to DNA damage.
NASP	Nuclear Autoantigenic Sperm Protein (Histone-Binding)	H1 histone binding protein that is involved in transporting histones into the nucleus of dividing cells. Required for DNA replication, normal cell cycle progression and cell proliferation.
MARK2	MAP/Microtubule Affinity-Regulating Kinase 2	
SF3A3	Splicing Factor 3a, Subunit 3, 60kDa	Subunit of the splicing factor SF3A required for A complex assembly formed by the stable binding of U2 snRNP to the branchpoint sequence (BPS) in pre-mRNA.
TRAM1	Translocation Associated Membrane Protein 1	Serine/threonine-protein kinase involved in cell polarity and microtubule dynamics regulation.
TRIM36	Tripartite Motif Containing 36	E3 ubiquitin-protein ligase which mediates ubiquitination and subsequent proteasomal degradation of target proteins. Involved in chromosome segregation and cell cycle regulation.
ACAA1	Acetyl-CoA Acyltransferase 1	This gene encodes an enzyme operative in the beta-oxidation system of the peroxisomes.
PNISR	PNN-Interacting Serine/Arginine-Rich Protein	A protein coding gene. GO annotations related to this gene include poly(A) RNA binding.

Figure S7. HM-associated AS genes are more lineage-specific. (A) The length of HM-associated AS exons are much shorter than that of HM-unassociated AS exons. p-values, student's t-test. **(B)** Venn graph shows the overlap of HM-associated AS genes across all cell lineages, indicating more lineage specificity compared with all AS genes shown in **Figure S3D, E**. **(C)** Venn graph shows the overlap of HM-associated AS genes across cell lineages excluding the IMR90. **(D)** Pairwise overlaps of HM-associated AS genes across cell lineages. The percentages (%) in parentheses show the ratio between the number of pairwise intersection and union, which were used to define the shading darkness. **(E)** The most common HM-associated AS genes shared by all lineages. The last two underlined genes are the additional ones not shared by IMR90.

Related to **Figure 4**.

A

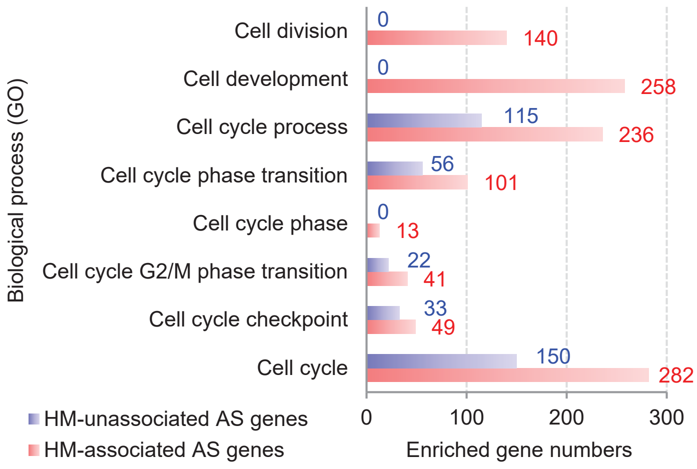

B

Lineages	p-value	FDR B&H adjustment	FDR B&Y adjustment	Bonferroni adjustment	Genes from input (# enriched/total input)	Genes in annotation
ME	2.808E-5	9.018E-3	7.833E-2	9.325E-2	40/224	1780
TBL	2.622E-6	2.701E-3	2.415E-2	1.127E-2	56/323	1780
NPC	7.343E-6	7.527E-3	6.836E-2	3.624E-2	81/540	1780
MSC	NA	NA	NA	NA	56/387	1780
IMR90	7.564E-12	1.782E-8	1.682E-7	5.346E-8	199/1340	1780

C

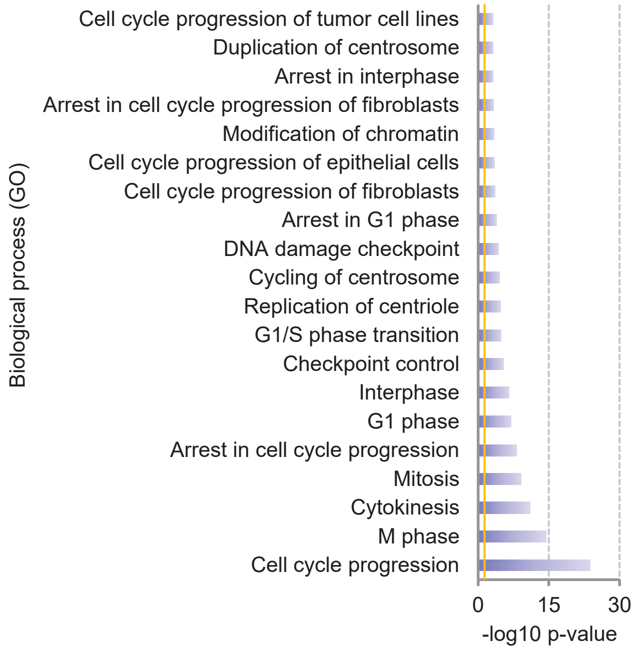

D

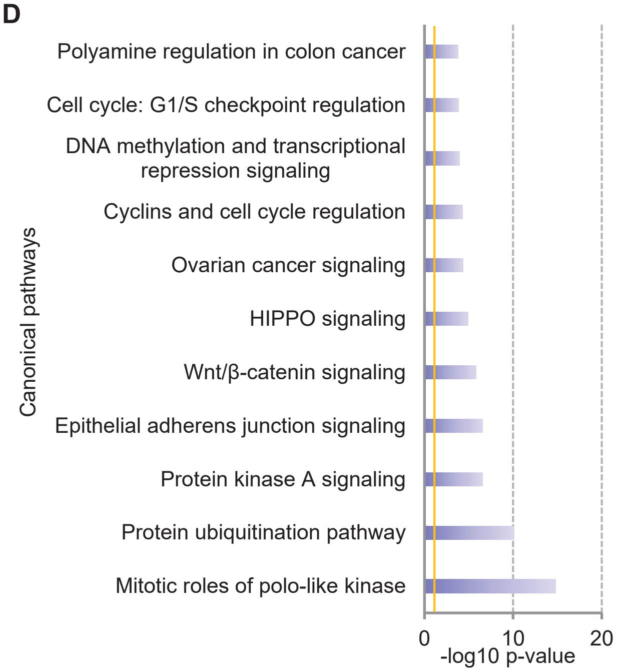

Figure S8. HM-unassociated AS genes are enriched in G1 cell-cycle phase and pathways for self-renewal. (A) Gene ontology (GO) enrichment analysis shows HM-associated AS genes are more enriched in cell-cycle progression than HM-unassociated AS genes, shown as the enriched gene numbers of each subgroup. **(B)** The enrichments in cell cycle of HM-associated AS genes are consistent across cell lineages, with the MSC as an exception. **(C)** HM-unassociated AS genes involved in cell-cycle progression prefer to function in G1 phase and cell-cycle arrest, shown as the -log10 p-values after FDR (≤ 0.05) adjustment. **d** The enriched canonical pathways show HM-unassociated AS genes are related more with G1 phase and Wnt/β-catenin signaling, which are important for self-renewal. The vertical lines (yellow) indicate the significance cutoff of 0.05.

Related to **Figure 4**.

A

Name	GeneID	Ensembl ID	CCDS ID	UniProt	RefSeq ID	mRNA length	Protein length	Protein sequence variation
PBX1a /<br>PBX1-001	5087	ENSG00000185630<br>ENST00000420696<br>ENSP00000405890	1246	P40424	NM_002585<br>NP_002576	6918nt	430aa	Canonical sequence<br>Full length: 1-430 aa
PBX1b /<br>PBX1-002	5087	ENSG00000185630<br>ENST00000367897<br>ENSP00000356872	55654	P40424	NM_001204961<br>NP_001191890	6805nt	347aa	334-347:<br>SSSSFNMSNSGDLF→GYPSPCYQPDRRIQ<br>348-430: missing<br>Full length: 1-347aa
PBX1c /<br>PBX1-020	5087	ENSG00000185630<br>ENST00000627490<br>ENSP00000405892	55653	P40424	NM_001204963<br>NP_001191892	4162nt	420aa	401-420: ANGGWQDATTPSSVTSPTEG →<br>HLPRHPRQAHYHFRLPTWHP<br>420-430: missing<br>Full length: 1-420aa

nt: nucleotide; aa: amino acid

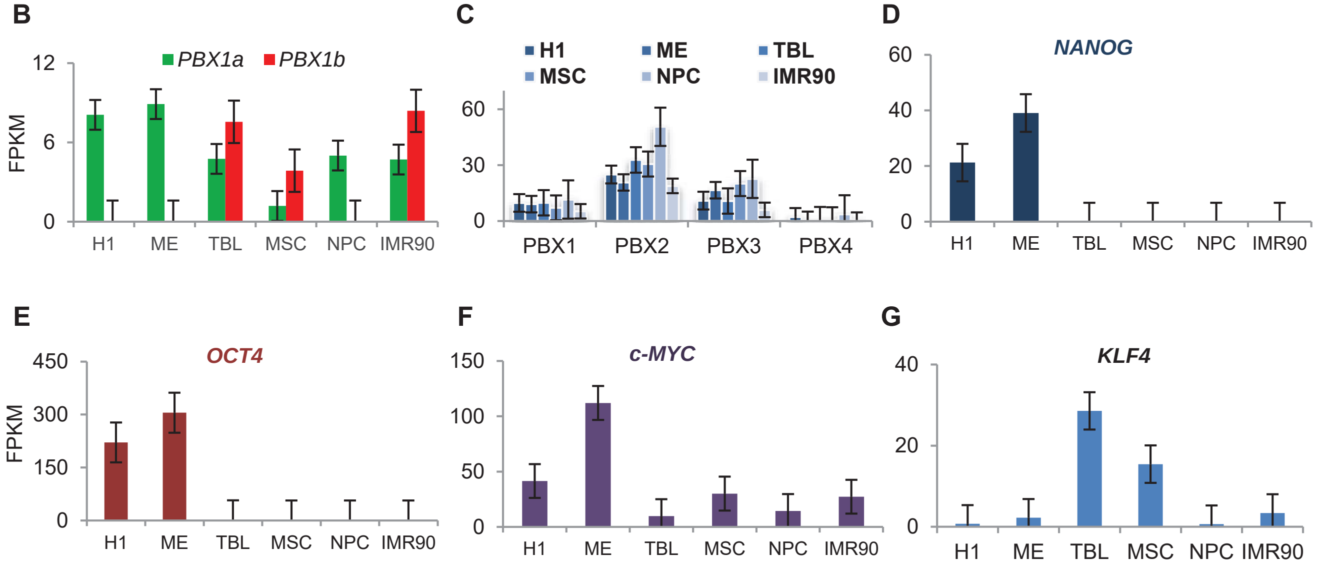

Figure S9. Isoform switch from PBX1a and PBX1b during hESC differentiation. (A) The sequence information of transcript and protein isoforms of PBX1. **(B)** The expression levels of isoforms *PBX1a* and *PBX1b* in hESCs and differentiated cells. The error bars represent standard error of the mean (SEM, n = 2). **(C)** The expression levels of PBX family genes show no significant difference across cell types (2-fold change as cutoff). **(D-G)** The expression levels of Yamanaka factors in six studied cell types.

Related to **Figures 5 and 6**.

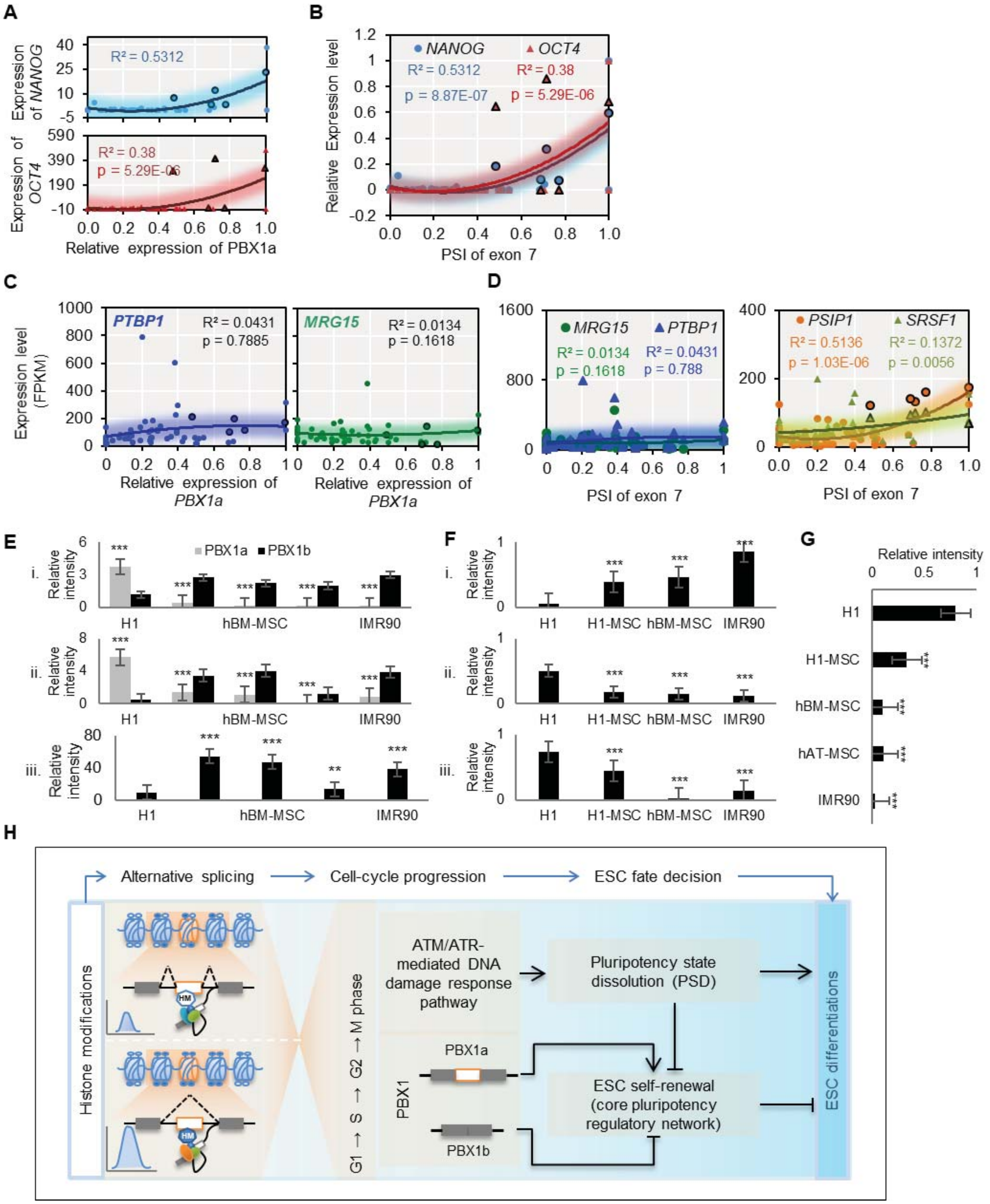

Figure S10. Isoform switch of PBX1 links H3K36me3 to hESC fate decision. (A) The expression levels of NANOG and OCT4 genes are significantly positive correlated with the expression of PBX1a, shown as the relative expression levels. **(B)** The expression levels of NANOG and OCT4 genes are significantly positive correlated with the PSI of exon 7 for PBX1. **(C)** No significant correlations between the expression levels of PTB/MRG15 and PBX1a are observed. **(D)** The correlations for the expression levels of *PTB/MRG15* (insignificant) and *PSIP1/SRSF1* (significant) with the inclusion level of exon 7 of PBX1. **(E)** The relative expressions of PBX1a and PBX1b based on bands in gel images of RT-PCR and western blotting's, represented as the relative band intensities normalized by bands of ß-Actin. Related to Figure 6B. **(F)** The relative signals of ChIP-PCR assays, represented as the relative band intensity *v.s.* the input bands. Related to Figure 6C i-iii. **(G)** The relative signals of RIP-PCR assays, represented as the relative band intensity *v.s.* the input bands. Related to Figure 6D. The error bars indicate the standard deviations of three replicates. Asterisks represent the significances based on ANOVA.**, p<0.01; ***, p<0.001. **(H)**The presented mechanism conveying the HM information into cell fate decision through the AS of cell-cycle factors or the core components of pathways that controlling cell-cycle progression.

Related to **Figures 5 and 6**.

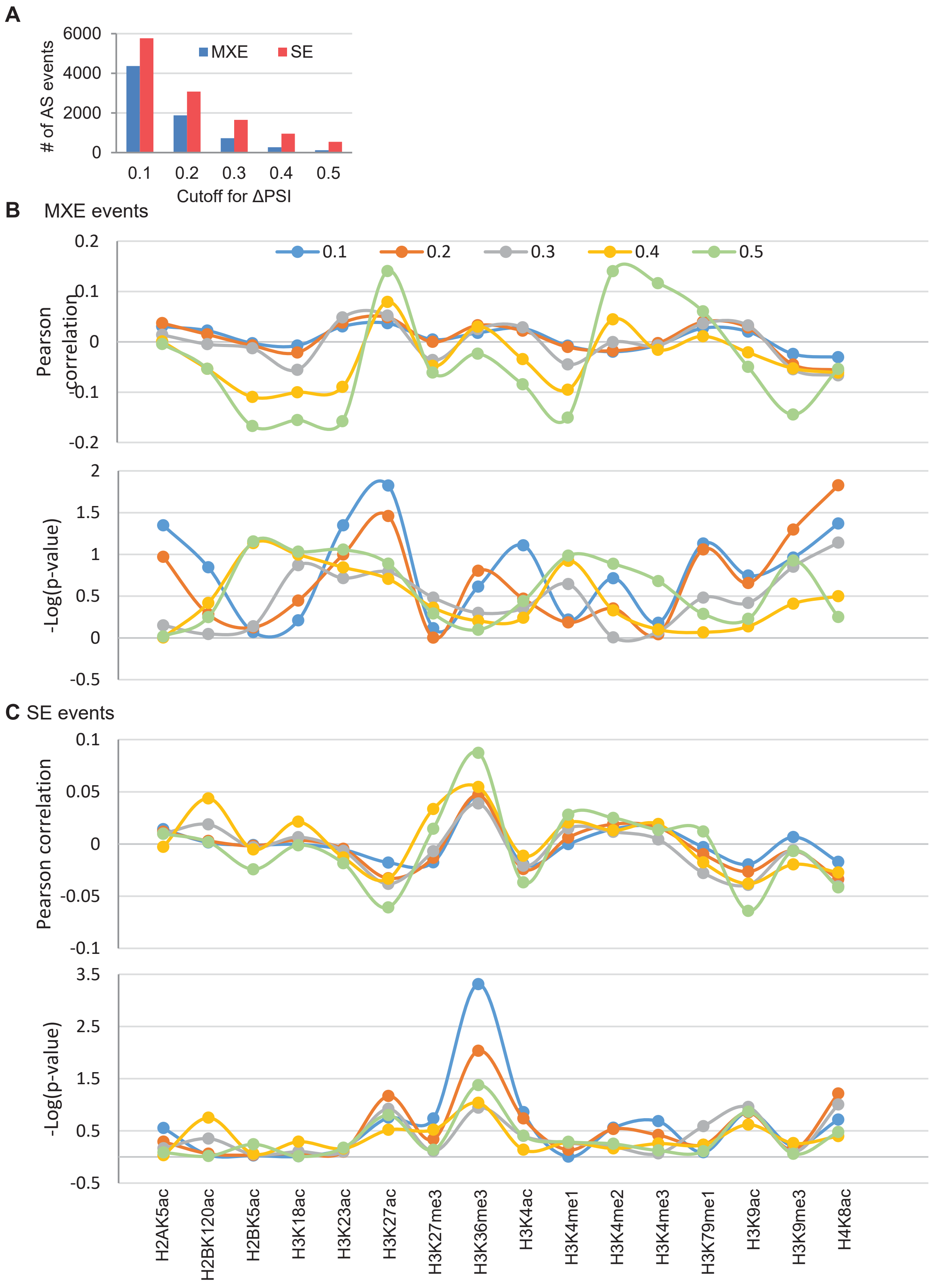

Figure S11. The effect of ΔPSI cutoffs for AS-HM correlations. (A) The number of identified AS events decrease significantly with increasing of the ΔPSI cutoff. (B) The effect of ΔPSI cutoffs for AS-HM correlations of MXE events. (B) The effect of ΔPSI cutoffs for AS-HM correlations of MXE events. The upper panels of (B) and (C) represent the Pearson correlation and lower panels represent the log p-values of correlation test using R function cor.test().

Table S1. The number of all AS events identified during hESC differentiation.

AS types	ME	TBL	MSC	NPC	IMR90
MXE	177	426	398	589	2583
SE	484	687	1152	1636	1655
A5SS	360	466	608	596	1124
A3SS	357	465	572	564	1108
ATSS	418	779	914	810	1221
ATES	364	640	803	702	935
IR	146	152	226	189	305
**Total**	2306	3615	4673	5086	8931

AS types: MXE, mutually exclusive exon; SE, skipping exon; A5SS, alternative 5’ splice site; A3SS, alternative 3’ splice site; ATSS, alternative transcription start site; ATES, alternative transcription end site; IR, intron retention.

Table S5. The PCR primers used in this study.

Gene name	Sequences	Product length	Assay
OCT4	F:GGAGGAAGCTGACAACAATGA<br>R:CTCTCACTCGGTTCTCGATACT	104 bp	qRT-PCR (Figure 6A)
SOX2	F:TGATGGAGACGGAGCTGAA<br>R:GGGCTGTTTTTCTGGTTGC	103 bp	qRT-PCR (Figure 6A)
NANOG	F:TGAAATCTAAGAGGTGGCAGAA<br>R:CCTGGTGGTAGGAAGAGTAAAG	108 bp	qRT-PCR (Figure 6A)
KLF4	F:ACCTACACAAAGAGTTCCCATC<br>R:ATCTGAGCGGGCGAATTT	108 bp	qRT-PCR (Figure 6A)
C-MYC	F:CTGAGGAGGAACAAGAAGATGAG<br>R:TGTGAGGAGGTTTGCTGTG	123 bp	qRT-PCR (Figure 6A)
ß-actin	F:GGATCAGCAAGCAGGAGTATG<br>R:AGAAAGGGTGTAACGCAACTAA	96 bp	qRT-PCR (Figure 6A)
PBX1	F:GCCAAGAAGAGTGGCATCACAGTC<br>R:CCCTGCGGACTGTACATCTGACT	386bp (PBX1a)<br>273bp (PBX1b)	RT-PCR (Figure 6B)<br>RIP-PCR (Figure 6D)
ß-actin	F:AGCCATGTACGTTGCTATCC<br>R:CGTAGCACAGCTTCTCCTTAAT	266 bp	RT-PCR (Figure 6B)
PBX1 exon7	F:CCCTTTATTGCTTGCTCTGTTC<br>R:CCCTGTGCTTACTGTTCTTCT	536 bp	ChIP-PCR (Figure 6C ii-iii)
NANOG promoter	F:TCCTTGGCGAAGAATGTAGTAAG<br>R:CCGGAGAAGATTAAGGATAGGG	400 bp	ChIP-PCR (Figure 6C-i)

All primer sequences are presented in 5'->3'.
